# Adaptation and validation of the financial stress scale in social conflict contexts: a study conducted with small and medium-sized Peruvian entrepreneurs

**DOI:** 10.3389/fpsyg.2023.1241005

**Published:** 2023-12-14

**Authors:** Madona Tito-Betancur, Mariné Huayta-Meza, Josué Turpo Chaparro, Wilter C. Morales-García, Oscar Mamani-Benito

**Affiliations:** ^1^Facultad de Derecho, Universidad Tecnológica, Universidad Tecnológica del Perú, Arequipa, Peru; ^2^Facultad de Ciencias Empresariales, Universidad Peruana Unión, Juliaca, Peru; ^3^Escuela de Posgrado, Universidad Peruana Unión, Lima, Peru; ^4^Escuela de Medicina Humana, Facultad de Ciencias de la Salud, Universidad Peruana Unión, Lima, Peru; ^5^Facultad de Teología, Universidad Peruana Unión, Lima, Peru; ^6^Sociedad Científica de Investigadores Adventistas (SOCIA), Universidad Peruana Unión, Lima, Peru; ^7^Facultad de Derecho y Humanidades, Universidad Señor de Sipán, Chiclayo, Perú

**Keywords:** financial stress, political conflicts, social conflicts, validation study, Peru

## Abstract

**Introduction:**

Social conflicts have repercussions on the mental health of the economically active population.

**Objective:**

To adapt and validate the Financial Stress Scale in the context of social conflicts (ESECPS).

**Method:**

An instrumental study involving 2,242 owners of small and medium enterprises (50.9% women), aged between 18 and 74 years old, selected through a non-probabilistic purposive sampling. The participants were recruited across three regions of Peru during periods of protests and strikes against the incumbent Peruvian government. The instrument for adaptation was the financial stress scale EFEmp-Cov19, created in the context of the COVID-19 pandemic’s impact.

**Results:**

All items proved to be clear, relevant, and representative (V > 0.70). Exploratory Factor Analysis (EFA) revealed the existence of one underlying factor across the 11 items (KMO = 0.962, Bartlett = 5434.3; df = 55; *p* < 0.001). However, for Confirmatory Factor Analysis (CFA), items 4 and 11 were removed, resulting in support for a unidimensional model with 9 items (*χ*2 = 262.73, df = 23, *p* < 0.001; RMR = 0.022; TLI = 0.972; CFI = 0.980; and RMSEA = 0.072). Regarding reliability, a very high value was found (*ω* = 0.92).

**Conclusion:**

The ESECPS demonstrates adequate psychometric properties, making it a suitable measure to assess financial stress among Peruvian entrepreneurs facing economic instability and financial threats in the context of social conflicts.

## 1 Introduction

Social conflicts negatively impact the economic growth of countries (de Groot et al., 2022). They are often the main cause of economic contraction, political instability, job loss, weakening business confidence, and deterioration of the investment climate (Paredes and Encinas, 2020; Wen et al., 2022).

In the Peruvian context, in recent years, intense social conflicts have occurred due to political events (Buschschlüter, 2022). The most recent began at the end of 2022, when Pedro Castillo (former president) was removed from office after he attempted to dissolve the Congress of the Republic. This event disturbed the mood of much of the population in the southern regions of Peru, with areas such as Arequipa, Apurímac, Ayacucho, Cuzco, and Puno initiating protests demanding new presidential elections and the closure of the Congress. Without a response from the succeeding government, these regions became scenes of constant roadblocks, protests against the succeeding government, and clashes between demonstrators and the National Police of Peru (Olmo, 2023), resulting in tragic outcomes such as the 18 people killed in Puno (BBC News Mundo, 2023).

As a result of these events, the population from regions with a higher presence of social conflicts began to experience alterations in their mental health (Valle and Contreras-Pizarro, 2023), especially the economically active population, who had to face financial threats and a negative perception of financial well-being (Dube, 2023). All this led to the weakening and closure of small and medium-sized enterprises, in some cases due to a lack of economic income to sustain themselves, and in others, for fear of being victims of looting and other threats by protesters.

### 1.1 Financial stress

For this reason, one of the main manifestations experienced by the economically active population is stress, a construct defined as an organism’s response to a situation perceived as threatening or challenging. Although current literature clarifies that there is still no unified theoretical framework to explain entrepreneurial stress, given the challenge of conceptually unifying the construct, and also that previous studies have not adequately captured the entire range of stressors from a business and psychological perspective (Arshi et al., 2021), the authors turn to the theoretical model proposed by Richard S. Lazarus and Judah Folkman (Biggs et al., 2017), which allows understanding and analyzing the stress generated by social conflicts.

According to this model, stress occurs when environmental demands exceed an individual’s resources, generating a process of cognitive and emotional assessment that can lead to adaptive or maladaptive coping responses. This is the case for small and medium-sized entrepreneurs, in whom financial stress arises due to decreased sales, economic instability, the threat of looting or damage, and severe difficulties in meeting financial and tax commitments. All these situations represent significant challenges for business owners and managers, who must face an uncertain and volatile environment, having to make critical decisions for the survival of their companies (Rashid and Ratten, 2020).

### 1.2 Literature review

Upon reviewing the available scientific literature, one finds sufficient reasons to determine the importance of continuing to investigate stress, in this case for financial reasons. Various studies have found that stressors have a negative impact on the health and well-being of the economically active population, especially psychosocial factors, which can alter mental health to the point of developing major disorders (Vigoda, 2002; Zuelke et al., 2020). This is extremely serious given that epidemiological and economic estimates suggest that the burden of mental disorders in terms of economic losses and health could be much greater than previously assessed. For example, an estimate by Arias et al. (2022) reveals that the global burden of mental disorders has an economic value of approximately 5 trillion dollars, and in underdeveloped regions like Africa, losses could represent 4% of the gross domestic product, while 8% in high-income regions such as North America.

In this context, it is necessary to have valid and reliable measures to assess stress. However, the instruments available in the literature and preferred by researchers, such as the Job Stress Questionnaire by Karasek et al. (1998), Occupational Stress Inventory of Osipow (1998), the Global Stress Perception Scale (Guzmán and Reyes-Bossio, 2018), the Brief Perceived Stress Scale (Campo-Arias et al., 2015), and even recently validated measures for social crisis contexts like the pandemic-related stress scale (Campo-Arias et al., 2020) and the financial stress scale for dependent (Carranza Esteban et al., 2021) and independent workers (Tito-Bentancur et al., 2021), do not address the specific concerns of business owners and managers in situations of social conflict.

### 1.3 Justification

Given this gap in the scientific literature, due to the limited specific information on this topic (Perrewé et al., 2000), there is a need for a scale that can measure financial stress in the context of social conflicts in owners and managers of small and medium-sized enterprises. Although referring to owner and manager implies recognizing that they are in completely different positions, the literature reveals that the psychological costs of owning and managing a small and medium-sized enterprise do not depend so much on the position, but on the individual capacity for adaptation and, more particularly, on the perception of control over the environment (Fernet et al., 2016).

Thus, a measure developed during the COVID-19 pandemic presents itself as an ideal alternative, with the objective of evaluating the perception of financial stress in small and medium-sized entrepreneurs. At the time, this measure managed to demonstrate content-based validity, construct validity through exploratory factor analysis, and reliability through the evaluation of internal consistency (Tito-Bentancur et al., 2021), and has even been used in other studies to measure the financial impact in contexts of health crisis in Peru, proving to have adequate psychometric performance (Mamani-Benito et al., 2021).

### 1.4 Objective

Based on all the above, the main objective was to adapt and validate the Financial Stress Scale in the context of social conflicts (ESECPS).

## 2 Method

### 2.1 Design

This study is instrumental (Ato et al., 2013), as it explores the main psychometric properties (content-based validity, internal structure, and reliability) of a documentary-type measure.

### 2.2 Participants

The population corresponds to the entirety of owners and managers of small and medium-sized enterprises (SMEs) in Peruvian territory. Given the limitations for physical access to the three regions of Peru (primarily due to roadblocks), a non-probabilistic sampling of an intentional type was decided upon. Inclusion criteria were applied as follows: being of legal age (over 18), owning or managing an SME, and completing the entire online form. Exclusion criteria included: being a salaried employee who also owns an SME, being a minor, and not providing informed consent.

Consequently, the study engaged the voluntary participation of 2,242 SME owners. The majority were women (50.9%), aged between 18 and 74 years (mean age = 31.99, SD = 11.41), predominantly single (56.7%), with a higher education level (72.8%), residing in cities within the mountain regions (76.3%), with formalized businesses (38.4%), and a larger representation from the commerce sector (40.7%) (Table 1).

**Table 1:** Sociodemographic characteristics of the total sample and subsamples.

Variables	Total (*N* = 2,242)		EFA (*N* = 500)		CFA (*N* = 1742)	
	*n*	%	*n*	%	*n*	%
Sex						
Male	1,101	49.1	228	45.6	873	50.1
Female	1,141	50.9	272	54.4	869	49.9
Marital status						
Married	428	19.1	108	21.6	320	18.4
Living together	459	20.5	67	13.4	392	22.5
Single	56.7	1,272	305	61	967	55.5
Divorced	2.1	48	11	2.2	37	2.1
Widowed	1.6	35	9	1.8	26	1.5
Education						
Primary	44	2.0	2	0.4	42	2.4
Secondary	548	24.4	64	12.8	484	27.8
Higher	1,632	72.8	434	86.8	1,198	68.8
No education	18	0.8	0	0	18	1
Residence						
Highlands	1710	76.3	232	46.4	1,478	84.8
Coast	448	20	245	49	203	11.7
Jungle	84	3.7	23	4.6	61	3.5
Formalization status						
Formalized	862	38.4	275	55	587	33.7
In process	761	33.9	123	24.6	638	36.6
Not formalized	619	27.6	102	20.4	517	29.7
Work sector						
Trade	913	40.7	140	28	773	44.4
Health	301	13.4	138	27.6	163	9.4
Transport	244	10.9	35	7	209	12
Food	229	10.2	51	10.2	178	10.2
Tourism	54	2.4	7	1.4	47	2.7
Others	501	22.3	129	25.8	372	21.3

*n*, frequency; %, Percentage.

### 2.3 Instrument

The base instrument for this adaptation corresponds to the Financial Stress Scale (EFEmp-Cov19) designed for Peruvian small and medium-sized entrepreneurs (Tito-Bentancur et al., 2021). This measure consists of 11 items distributed in a single factor, with five response options: strongly disagree, disagree, indifferent, agree, and strongly agree. In the validation study, it demonstrated content-based validity through the Aiken’s V coefficient, where it obtained values above 0.70, internal structure-based validity through exploratory factor analysis, and reliability through Cronbach’s Alpha coefficient (> 0.80).

### 2.4 Procedure

The study was conducted in stages. It began with the linguistic and contextual adaptation of the items, a process led by three of the authors, headed by the lead researcher, who has experience in the business field. Second, once a consensus was reached among the authors, the scrutiny of seven experts was requested (three university professors specializing in human resources and business development, two researchers certified by the National Council of Science and Technology of Peru, and two businessmen from the commerce and tourism sector in the Puno region, Peru), who evaluated the clarity, representativeness, and relevance of the items through a validation format proposed by Ventura-León (2019) guiding to assess the degree of validity under Aiken’s V coefficient. Third, a focus group was organized, in which 25 business owners from the Puno department participated, who were presented with the 11 items, asking for their opinion regarding the wording and possible conflicts in understanding the items. As a result of this process, minor observations were made and corrected by two of the researchers.

Fourth, the scale was initially applied to 500 small and medium-sized entrepreneurs, and an exploratory factor analysis was carried out. Then, it was applied to another 1742 small and medium-sized entrepreneurs for confirmatory factor analysis. In this case, data collection was carried out virtually. To this end, a questionnaire was created using Google Forms, which remained active from March 12 to April 8, 2023. The link to this tool was shared via Facebook, Instagram, and WhatsApp, through an invitation targeted at working adults who are owners and/or managers of small or medium-sized businesses. On Facebook and Instagram, six sets of ads were run, reaching 87,231 people identified as independent workers within our targeted segment. In the case of WhatsApp, it was managed through direct contacts in companies within the commerce, health, transportation, food, and tourism sectors, primarily. Before starting the questionnaire, participants gave their consent through an informed consent form, which explained the purpose of the study, emphasizing that participation was voluntary, anonymous, and confidential.

### 2.5 Data analysis

The analysis was conducted in four stages; first, the descriptive statistics (mean, standard deviation, skewness, and kurtosis) of the 11 items of the scale were analyzed. The criterion used for skewness and kurtosis was > ± 1.5 (Peréz and Medrano, 2010). In the second phase, the general sample was divided (500 cases for exploratory factor analysis and 1742 for confirmatory factor analysis). For conducting an EFA, adherence to parameters according to the Kaiser-Meyer-Olkin (KMO) and Bartlett’s test was considered. Once verified, the software was configured using the unweighted least squares estimation method with Promax rotation. In the third stage, a Confirmatory Factor Analysis (CFA) was executed, and structural equation modeling (SEM) was considered. To evaluate the goodness-of-fit measures, the Goodness of Fit Index (GFI), Tucker-Lewis Index (TLI), Comparative Fit Index (CFI) were used. The parameters for the Root Mean Square Error of Approximation (RMSEA) and the Root Mean Square Residual (RMR) were also considered, following the criteria recommended by Hu and Bentler (1999), who suggest that GFI, TLI, and CFI should be higher than 0.90, and RMSEA and SRMR should be less than 0.08. Descriptive analyses and EFA were conducted using FACTOR Analysis version 10.1. To perform CFA, AMOS version 21 statistical software was used, and the SPSS version 25.0 statistical program was used to calculate the scale’s reliability.

### 2.6 Ethical considerations

The research project was reviewed by the ethics committee of the Peruvian Union University, which approved it under an official document with number: 2023-CEUPeU-017.

## 3 Results

In Table 2, the adapted items from the FSSCPSC are observed. In terms of content validity, items 2, 3, 4, 5, 7, 8, 9, 11 prove to be the clearest (*V* = 1, 95% CI: 0.80–1.00), items 1, 4, 5, 6, 8, 9, 10, 11 appear as more representative (*V* = 1, 95% CI: 0.80–1.00), and items 2, 3, 5, 7, 9, 11 turned out to be more relevant (*V* = 1, 95% CI: 0.80–1.00).

**Table 2:** Adaptation of the ESECPS/FSSSCC items from the EFEmp-Cov19 scale and Aiken’s V indicators (*n* = 7 experts).

EFEmp-Cov19	ESECPS (Spanish version)	FSSSCC (English version)	Clarity		Representativeness		Relevance	
			*V*	IC 95%	*V*	IC 95%	*V*	IC 95%
I feel insecure about the profitability of my company.	Me frustra ver como en mi empresa/negocio disminuyen las ventas/servicios	It frustrates me to see the sales/services in my company/business decline.	0.93	0,70 – 0,99	1	0,80 – 1,00	0.93	0,70 – 0,99
In case of bankruptcy, it would be difficult for me to recover.	Me preocupa que mi empresa/negocio no se pueda reactivar si se suspenden las actividades económicas	I’m concerned that my company/business will not be able to reactivate if economic activities are suspended.	1	0,80 – 1,00	0.93	0,70 – 0,99	1	0,80 – 1,00
I fear/worry about over-indebting myself to save my company.	Tengo miedo/preocupación que saqueen la mercadería y/o dañen las herramientas de trabajo de mi empresa/negocio.	I fear/worry about looting of merchandize and/or damage to the work tools of my company/business.	1	0,85 – 1,00	0.93	0,70 – 0,99	1	0,85 – 1,00
The country’s economic instability has affected the productivity/sales of my company.	El conflicto político y social afecta la productividad/ventas de mi empresa/negocio.	The political and social conflict affects the productivity/sales of my company/business.	1	0,85 – 1,00	1	0,80 – 1,00	0.93	0,70 – 0,99
I am concerned that the health crisis may alter the financial stability of my company.	Me preocupa que las constantes manifestaciones alteren la estabilidad financiera de mi empresa/negocio.	I am concerned that constant demonstrations will alter the financial stability of my company/business.	1	0,85 – 1,00	1	0,85 – 1,00	1	0,85 – 1,00
The borrowing capacity of my company has been affected by the pandemic.	Debido al cierre de los negocios me preocupa no poder cumplir con compromisos financieros de mi empresa/negocio	Due to the closure of businesses, I am concerned about not being able to fulfill the financial commitments of my company/business.	0.93	0,70 – 0,99	1	0,85 – 1,00	0.93	0,70 – 0,99
I worry about losing the capital invested in my company.	Ante las constantes paralizaciones me preocupa perder el capital invertido en mi empresa/negocio	Given the constant shutdowns, I am concerned about losing the capital invested in my company/business.	1	0,85 – 1,00	0.93	0,70 – 0,99	1	0,85 – 1,00
I fear/worry about losing suppliers for my company.	Ante el bloqueo de carreteras tengo miedo/preocupación que la mercadería no llegue a tiempo a mi negocio/empresa.	With the blocking of roads, I fear/worry that merchandize may not arrive on time at my business/company.	1	0,85 – 1,00	1	0,80 – 1,00	0.93	0,70 – 0,99
I fear/worry about losing potential customers for my company.	Tengo miedo/preocupación de perder clientes potenciales a consecuencia de constantes huelgas.	I fear/worry about losing potential customers due to constant strikes.	1	0,85 – 1,00	1	0,85 – 1,00	1	0,85 – 1,00
I fear/worry about not being able to cover the expenses of my company’s staff.	Me preocupa que debido al cierre constante de mi empresa/negocio no pueda cumplir con los salarios de mis trabajadores	I am concerned that due to the constant closure of my company/business, I may not be able to pay my workers’ salaries.	0.93	0,70 – 0,99	1	0,85 – 1,00	0.93	0,70 – 0,99
I worry about not being able to meet tax obligations.	La inestabilidad económica durante la crisis política y social hace que no pueda cumplir con mis obligaciones tributarias	The economic instability during the political and social crisis prevents me from fulfilling my tax obligations.	1	0,80 – 1,00	1	0,80 – 1,00	1	0,80 – 1,00

Table 3 shows the mean, standard deviation, skewness, and kurtosis of the 11 items. It is observed that item 7 has the highest average score (*M* = 3.76) and item 1 shows the greatest dispersion (SD = 1.70). Skewness and kurtosis scores for the items are within acceptable values (As </= 2, K </= 7). On the other hand, it is appreciated that all the items on the scale present corrected item correlation coefficients with the total elements above 0.30.

**Table 3:** Descriptive statistics for the ESECPS scale (*n* = 2,242).

Items	*M*	SD	As	*K*	i1	i2	i3	i4	i5	i6	i7	i8	i9	i10	i11
i1	3.49	1.70	−0.68	−0.66	1										
i2	3.42	1.51	−0.52	−0.77	0.61	1									
i3	3.63	1.60	−0.80	−0.38	0.66	0.60	1								
i4	3.72	1.51	−0.95	−0.03	0.68	0.68	0.72	1							
i5	3.75	1.44	−1.04	0.23	0.65	0.62	0.69	0.74	1						
i6	3.74	1.40	−0.95	0.08	0.65	0.60	0.73	0.74	0.80	1					
i7	3.76	1.42	−0.93	0.04	0.62	0.60	0.67	0.70	0.76	0.79	1				
i8	3.70	1.52	−0.85	−0.20	0.63	0.60	0.73	0.71	0.70	0.73	0.74	1			
i9	3.73	1.45	−0.94	0.04	0.65	0.64	0.71	0.71	0.75	0.78	0.78	0.81	1		
i10	3.71	1.48	−0.89	−0.10	0.61	0.59	0.71	0.71	0.71	0.78	0.75	0.75	0.82	1	
i11	3.69	1.54	−0.84	−0.26	0.60	0.59	0.69	0.69	0.72	0.76	0.76	0.73	0.78	0.85	1

*M*, Mean; SD, Standard Deviation; As, Skewness; *K*, Kurtosis.

After analyzing the Kaiser-Meyer-Olkin index (KMO = 0.962) and Bartlett’s test (5434.3, df = 55, *p* < 0.001), which yielded satisfactory results, an EFA was conducted. The unweighted least squares method with promin oblique rotation was used, and for factor determination, parallel analysis was used, which revealed the existence of a single factor underlying the 11 items. The rotated solution explains 77.10% of the total explained variance. All items present saturations greater than 0.40 (Table 4).

**Table 4:** Factor analysis of the ESECPS scale (*n* = 500).

Items	F1	h2
i1	0.75	0.56
i2	0.72	0.52
i3	0.82	0.68
i4	0.84	0.71
i5	0.85	0.73
i6	0.88	0.78
i7	0.86	0.74
i8	0.85	0.73
i9	0.89	0.80
i10	0.87	0.76
i11	0.86	0.74

F1, Single factor; h2, Communalities.

**Figure 1:**
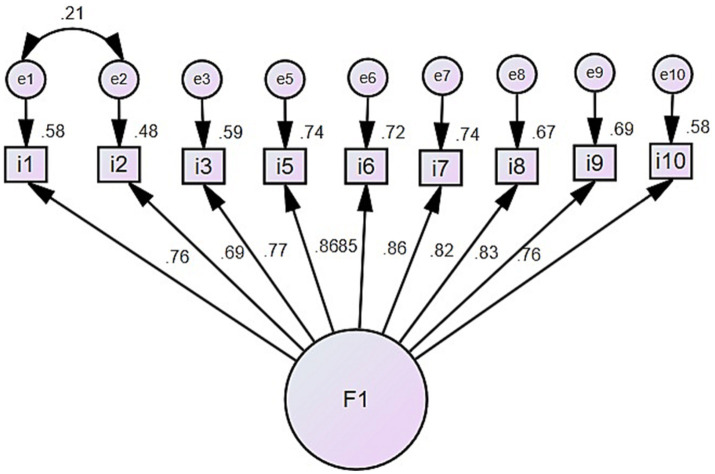
Factorial structure of the FSSCPSC scale.

To confirm the underlying model from the EFA, a CFA was executed to examine the internal structure of the scale. This was done after correlating errors (e1 and e2) and removing items 4 and 11, which, according to the authors’ understanding, could cause confusion in interpreting the construct. The structural model was validated, showing good fit indices as seen in Table 5 (*χ*2 = 262.73, df = 23, *p* < 0.001; RMR = 0.022; TLI = 0.972; CFI = 0.980; and RMSEA = 0.072). In summary, the 9-item, single-factor model was satisfactory (Figure 1).

**Table 5:** Goodness-of-fit indices for the factorial model of the ESECPS scale (*n* = 1742).

Model	*χ* ^2^	gl	*p*	TLI	CFI	RMSEA	CMIN/DF	RMR
Original	262.73	26	< 0.001	0.972	0.980	0.072	10.10	0.022

Finally, reliability was assessed through the method of internal consistency. For this purpose, the index was calculated using the Omega coefficient, which reported a value of *ω* = 0.92, indicative of a good level of scale reliability.

## 4 Discussion

The economy of a country can be directly affected by conflicts that occur within its own territory (Polachek and Sevastianova, 2012). Likewise, these conflicts, whether social or political, can be considered a demand that increases the risk of stress in individuals (Zuelke et al., 2020). In Latin America, institutional crises are common and are characterized by levels of conflict and power struggles (Helmke, 2017). Peru, for example, is a country with a weak political system (Paredes and Encinas, 2020), where studies report levels of stress related to the COVID-19 pandemic (Mamani-Benito et al., 2021), with negative effects on the Peruvian economy (Durst et al., 2021). In this sense, the objective of this research was to adapt and validate the Financial Stress Scale in Social Conflict Contexts (FSSSCC) - Escala de Estrés Financiero en Contextos de Conflictos Sociales (ESECPS) in Spanish.

The findings of this study revealed the robust psychometric attributes of the FSSSCC, which measures financial stress in social conflict contexts with 9 items in a single factor. Thus, an important finding of this investigation is a rigorous methodological procedure. Initially, experts assessed the performance and quality criteria considering three standards: representativeness, clarity, and sufficiency. The assessment was qualitative, as comments led to the inclusion and restructuring of performance criteria, allowing for better construct management. Furthermore, all items received a positive evaluation (Aiken, 1980).

Although, as a result of the EFA, the instrument included 11 items rated based on item acceptance, it was decided to eliminate items 4 “Political and social conflict affects the productivity/sales of my company/business” and 11 “Economic instability during the political and social crisis prevents me from fulfilling my tax obligations.” These were removed as they suggested the construct would encompass two aspects that should be analyzed independently (social conflict and political conflict). In this case, the authors focus solely on social conflict (which, according to the conflict relationships, occurs between the state and the population) (Instituto de Ciencias Hegel, 2021). Consequently, for the CFA, only 9 items were included, which satisfactorily adjusted in a unidimensional model, with factor loadings ranging from a minimum of 0.69 to 0.86. This aligns with the primary theoretical model that underpins this scale’s construction (Tito-Bentancur et al., 2021). Moreover, the FSSSCC/ ESECPS reliability coefficient value was satisfactory (Streiner, 2003) and acceptable (Hill and Hill, 2012), in line with what has been reported by other studies (Heo et al., 2020; Cardona-Montoya et al., 2022).

This research represents a significant contribution as it is the first validated version that can be used to measure financial stress in social conflict contexts among small and medium-sized business owners, which is similar to other instruments on financial stress in independent workers featuring a unifactorial model (Carranza Esteban et al., 2021) or the Financial Stress in Small and Medium-sized Business Owners (Tito-Bentancur et al., 2021), but different from other studies that contain up to three dimensions (Heo et al., 2020). In this sense, the relevance of the adaptation performed revolves around understanding that small and medium-sized business owners largely depend on the country’s economic stability, and even more so considering the recent collateral effects of the global COVID-19 pandemic (Ratten, 2020). In addition, during a crisis, an entrepreneur may sacrifice short-term gain for long-term survival (McMullen and Shepherd, 2006), although small and medium-sized business owners may also improvise to make the most of the current condition (Kraus et al., 2020).

The value of this research lies in the possibility of conducting future investigations about the consequences of social conflicts resulting from unexpected political events, especially in Latin American countries, where there is a certain weakness in governments. This situation is exploited by community groups who take measures such as blockades and strikes, resulting in negative repercussions on the economy (Zhang, 2023). Therefore, by determining the level of financial stress, one can infer and analyze factors that might cause problems in the emotional health of the working population.

This research also reveals other important implications. Firstly, this 9-item scale found a suitable distribution within a single factor. Because of its brevity, it can be quickly applied to the target population. Among its items, it assesses the financial stress of small and medium-sized business owners in a context of social conflict resulting from unexpected political events. In this regard, the initial items evaluate the frustration and economic losses due to the occurrence of strikes or social conflicts. Recent studies highlight the history of social, internal, and political conflicts that Peru has experienced for decades (Duche-Pérez et al., 2023), which are similar to those in other countries (Muoneke et al., 2023), making these items particularly relevant. Moreover, as research lines strengthen, the ESECPS instrument can highlight specific areas for the implementation of programs that assist small and medium-sized business owners in mitigating the consequences of political or social conflicts and thus reduce the level of financial stress (Ryu and Fan, 2023).

Despite its intriguing results, this research has some limitations. First, the type of sampling used is a concern, especially since over 70% of the participants are business owners from the Peruvian highlands, which could hinder the generalization of the results. Therefore, we recommend that in future applications, greater emphasis be given to other geographical regions of Peru to confirm the findings of this study. Second, this research involved both owners and managers of small and medium-sized enterprises, which may lead to conflicts when interpreting the psychometric performance of the ESECPS, given the distinctly different positions; hence, future studies should analyze whether the test is invariant across demographic variables such as gender and position within the company. Finally, data collection was virtual, which could result in some differences from data collected in person.

Despite these limitations, we consider this research a contribution to the literature on financial stress, especially in the context of political and social conflict. In conclusion, the FSSSCC/ESECPS scale is a validated and reliable instrument for measuring financial stress in political and social contexts among small and medium-sized Peruvian entrepreneurs.

## Data availability statement

The data analyzed in this study is subject to the following licenses/restrictions: the raw data supporting the conclusions of this article will be made available by the authors, without undue reservation. Requests to access these datasets should be directed to mamanibe@uss.edu.pe.

## Ethics statement

The studies involving humans were approved by ethics committee of the Peruvian Union University, which approved it under an official document with number: 2023-CEUPeU-017. The studies were conducted in accordance with the local legislation and institutional requirements. The participants provided their written informed consent to participate in this study.

## Author contributions

MT-B and OM-B: substantial contributions to the conception or design of the work, drafting the work or revising it critically for important intellectual content, provide approval for publication of the content. MH-M, WM-G, and JC: the acquisition, analysis, or interpretation of data for the work, drafting the work or revising it critically for important intellectual content provide approval for publication of the content. MT-B, OM-B, MH-M, WM-G, and JC: agree to be accountable for all aspects of the work in ensuring that questions related to the accuracy or integrity of any part of the work are appropriately investigated and resolved. All authors contributed to the article and approved the submitted version.
